# A Multicentric, Retrospective Efficacy and Safety Study of Nanosomal Docetaxel Lipid Suspension in Metastatic Castration-Resistant Prostate Cancer

**DOI:** 10.1155/2020/4242989

**Published:** 2020-11-24

**Authors:** Aseem Samar, Srikant Tiwari, Sundaram Subramanian, Nisarg Joshi, Jaykumar Sejpal, Mujtaba A. Khan, Imran Ahmad

**Affiliations:** ^1^Bhagwan Mahaveer Cancer Hospital and Research Centre, Jaipur, Rajasthan 302017, India; ^2^Jawaharlal Nehru Cancer Hospital & Research Centre, Bhopal, Madhya Pradesh 462001, India; ^3^VS Hospital, Madras Cancer Institute, Advanced Cancer Care, Chennai, Tamil Nadu 600031, India; ^4^Medical Affairs and Clinical Development, Intas Pharmaceuticals Ltd. Sola, Ahmedabad, Gujarat 380054, India; ^5^Jina Pharmaceuticals Inc., Libertyville, Illinois 60048, USA

## Abstract

**Purpose:**

To evaluate the efficacy and safety of nanosomal docetaxel lipid suspension (NDLS, DoceAqualip) in patients with metastatic castration-resistant prostate cancer (mCRPC).

**Materials and Methods:**

In this multicenter, retrospective study, we analyzed the medical charts of mCRPC patients, who were treated with NDLS administered as 2-weekly (50 mg/m^2^) or 3-weekly regimens (75 mg/m^2^). The study endpoints were prostate-specific antigen (PSA) response (>50% PSA decline from baseline), PSA progression (PSA increase from baseline beyond 12 weeks: ≥25% and ≥2 ng/mL), median PSA decline, and time-to-treatment failure (TTF). Overall survival (OS) and safety were also evaluated.

**Results:**

Data of 24 patients with mCRPC were analyzed in this study. NDLS was administered as a 2-weekly regimen in 37.5% (9/24; all first-line) patients and as a 3-weekly regimen in 62.5% patients (15/24; first-line: 20% (3/15), second-line: 80% (12/15)). Overall, PSA response was reported in 66.7% (16/24) patients. The PSA response was 77.8% (7/9 patients) in the 2-weekly group and 60% (9/15 patients) in the 3-weekly group. The median decline in PSA was 96.31% in the 2-weekly group and 83.29% in the 3-weekly group; the median TTF was 6.7 and 6.5 months in the 2 weekly group and 3-weekly group, respectively. The median OS was 14.6 months (follow-up: 5.5–25.8 months) in the 2-weekly group whereas it was not reached in the 3-weekly group (follow-up: 7.9–15.6 months). The most common hematological AEs were anemia, lymphopenia, thrombocytopenia, and neutropenia whereas nausea, weakness, constipation, vomiting, and diarrhea were the most common (≥10%) nonhematological AEs. Overall, NDLS treatment was well tolerated without any new safety concerns.

**Conclusions:**

Nanosomal docetaxel lipid suspension (2-weekly or 3-weekly) was effective and well tolerated in patients with metastatic castration-resistant prostate cancer.

## 1. Introduction

Prostate cancer is the second most common malignancy in men globally (1,276,106 new cases; 7.1% of all cancer cases) with the seventh highest cancer-related mortality (358,989 deaths, 3.8% of all cases) as per GLOBOCAN 2018 data [1]. Androgen-deprivation therapy (ADT), which includes bilateral orchiectomy or medical castration with gonadotropin-releasing hormone analogues, has been the cornerstone for the management of advanced prostate cancer, which can provide palliation of symptoms and improves patient survival [2].

Despite initial favorable response with ADT, the disease progresses to castration-resistant prostate cancer (CRPC) in almost all patients [3]. Approximately 10–20% patients in the Western countries present with metastatic disease, wherein for others, the disease metastasizes despite surgery or radiotherapy [4]. On the contrary, a majority (∼85%) of the patients with prostate cancer in India are diagnosed at advanced stages, maybe because of low awareness and limited access to screening, detection, and diagnostics [5].

Docetaxel was the first systemic agent to show survival advantage in mCRPC patients based on the pivotal Phase III TAX327 [4] and SWOG9916 [6] studies, and is considered as the standard first-line chemotherapy regimen [7]. Docetaxel has also demonstrated effectiveness and tolerability as second-line chemotherapy in the management of mCRPC [8]. Docetaxel is generally administered at 75 mg/m^2^ dose as 3-weekly cycles, but due to its tolerability and toxicity issues, weekly [9] and 2-weekly [10] regimens have also been evaluated in CRPC, which have shown tolerability advantages over the 3-weekly dosing schedule.

The formulation vehicles, polysorbate 80 and ethanol, used in the conventional docetaxel formulation, may be related to toxicity issues such as acute hypersensitivity reactions, cumulative fluid retention, peripheral neuropathy, severe nonimmunologic anaphylactoid reactions, infusion-site reactions, and alcohol intoxication [11–15]. Corticosteroid and antihistamine premedications are generally given to limit these toxicities; however, they are still observed [16]. To overcome these safety issues, a novel formulation of docetaxel, ‘nanosomal docetaxel lipid suspension (NDLS, DoceAqualip)', which is devoid of polysorbate 80 and ethanol, was developed [17]. NDLS is approved in India for the treatment of patients with advanced gastric adenocarcinoma, locally advanced or metastatic breast cancer (MBC) after failure of prior chemotherapy, non-small-cell lung cancer after failure of prior chemotherapy, for the induction treatment of locally advanced squamous cell carcinoma of the head and neck (LA SCCHN), and for the treatment of androgen-independent (hormone refractory) metastatic prostate cancer (HRPC).

Several studies have evaluated the efficacy and tolerability of NDLS in the treatment of breast, gastric, HRPC, non-small-cell lung cancer, ovarian, cervical, penile, and sarcoma patients [18–22]. We report, here, a multicenter, retrospective experience evaluating the efficacy and safety of NDLS in the treatment of mCRPC.

## 2. Materials and Methods

### 2.1. Study Design

In this multicenter, retrospective study, we analyzed the medical charts of adult men who were treated with NDLS as part of their routine clinical care for mCRPC and followed up from March 2017 to September 2019. The study endpoints were prostate-specific antigen (PSA) response as per the Prostate Cancer Clinical Trials Working Group (PCWG2) recommendations [23], defined as >50% decline in PSA levels from baseline; PSA progression, defined as ≥25% increase and ≥2 ng/mL increase from baseline beyond 12 weeks; median PSA decline from baseline to nadir; time-to-treatment failure (TTF), defined as time of NDLS initiation to the discontinuation due to any reason; and overall survival (OS), defined as time from treatment to death due to any cause; for patients who were still alive at the time of data analysis or who were lost to follow-up, OS was censored at the last recorded date that the patient was known to be alive.

Incidence of adverse events (AEs) documented in the treatment charts were recorded and graded according to the National Cancer Institute Common Terminology Criteria for Adverse Events (CTCAE) Criteria version 5.0. Similarly, data on deaths and discontinuations were captured from the patients' health records.

### 2.2. Ethics Statement

The study was reviewed and approved by the OM Ethics Committee (Ahmedabad, India). The study was conducted in accordance with the ethical principles that have their origin in the Declaration of Helsinki and in accordance with the International Conference on Harmonization's Good Clinical Practice guidelines (ICH-GCP), applicable regulatory requirements. Patient consent to review their medical records was not required by the ethics committee as this was a retrospective study and NDLS is already approved in India. Patient confidentiality was completely maintained as patient data were anonymized and no patient identifiers were used.

### 2.3. Statistical Analyses

Demographic and baseline characteristics were summarized using descriptive statistics. Categorical variables were summarized with frequency and percentage. Continuous variables were summarized with count, mean, standard deviation, median, minimum, and maximum. Response rate was presented as frequency and percentage of patients. The *χ*^2^ test was used to compare the distribution of patients in each category. Survival analysis was performed to measure lifetime or the length of time until the occurrence of an event (death in case of overall survival). Survival data were analyzed using a nonparametric procedure performed on PROC LIFETEST of SAS (Version 9.4) to measure the duration of time until a specified event occurs. The AEs were summarized as frequencies and percentages by the type of reactions.

## 3. Results

### 3.1. Patients Disposition and Demographics

Data of 24 patients with mCRPC, who were treated with NDLS, were retrospectively analyzed. The median age of the patients was 68 years (range: 48–83 years). The baseline characteristics of these patients are summarized in Table 1.

NDLS was administered as 50 mg/m^2^ in 2-weekly or 75 mg/m^2^ in 3-weekly cycles as a 1-hour infusion. In the 2-weekly group, NDLS was administered as a first-line chemotherapy in all 9 patients. In the 3-weekly group, NDLS was administered as first- and second-line chemotherapy in 20% (3/15) and 80% (12/15) patients, respectively. The median cumulative dose of NDLS was higher in the 2-weekly group versus the 3-weekly group (650 vs. 500 mg/m^2^). Patients in the 2-weekly group received a greater median number of NDLS chemotherapy cycles as compared to the 3-weekly group (14 vs. 10 cycles, respectively). The median actual dose intensity (21.04 vs. 18.75 mg/m^2^/week) and relative dose intensity (84% vs. 75%) were also higher for the 2-weekly group versus the 3-weekly group.  Table 2 provides the details of NDLS dose used in this study. Zoledronic acid (4 mg IV every 28 days) was administered in 91.7% (22/24) patients; all these patients had bone metastasis. Dexamethasone as premedication was administered in 75% (18/24) patients. Granulocyte-colony stimulating factor (GCSF) was used in all patients as primary prophylaxis.

### 3.2. Efficacy

**Figure 1:**
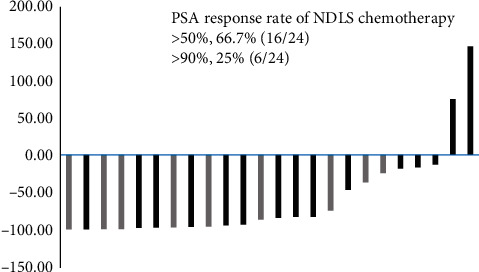
PSA response rate of NDLS chemotherapy. Bar charts show PSA response rate for each patient who received NDLS chemotherapy. Bars in black color indicate the 3-weekly group, and bars in grey color indicate the 2-weekly group.

The PSA response (>50% decrease in PSA) was achieved in 16 of 24 patients (66.7%) with NDLS chemotherapy. Furthermore, a decline in PSA >90% was reported in 6 patients (25%). The PSA response rate of NDLS chemotherapy is presented in Figure 1. The bars in black color indicate the 3-weekly group, and bars in grey color indicate the 2-weekly group. Abiraterone was the most common agent used after NDLS therapy (Table 3).

### 3.3. Time to Treatment Failure and Overall Survival

The median TTF was 6.7 and 6.5 months, for the 2-weekly and 3-weekly groups, respectively. Overall, patient survival data were collected from the date of administration of the first dose of NDLS-based therapy till the last follow-up date (September 2019) for alive patients and date of death for patients who died. In the 2-weekly group, only one out of nine patients was alive at the last follow-up (11.1%) and the median OS was 14.6 months (follow-up duration: 5.5–25.8 months). All patients in the 3-weekly group were alive at the last follow-up and the median OS was not reached (follow-up duration: 7.9–15.6 months).

### 3.4. Safety

At least, 1 AE was reported in 95.8% (23/24) patients. Grade 1 AEs were reported in 95.8% (23/24) patients, grade 2 AEs in 20.8% (5/24), and grade 3 AEs in 16.7% (4/24). Anemia, lymphopenia, thrombocytopenia, and neutropenia were the hematological AEs reported while nausea, vomiting, weakness, constipation, and diarrhea were the most common nonhematological AEs reported. None of the patients reported grade IV AE (Table 4).

## 4. Discussion

Several guidelines recommend the use of docetaxel with concurrent steroid (dexamethasone or prednisone) as the first- or second-line treatment for mCRPC. Docetaxel in combination with prednisone every 3 weeks is the preferred first-line therapy for mCRPC [24]. Furthermore, 2-weekly or weekly cycles of docetaxel have also been evaluated for the management of mCRPC [7, 9, 10]. We report, here, a multicenter, retrospective, real world experience on the effectiveness and tolerability of novel NDLS formulation in patients with mCRPC.

The landmark Phase III TAX327 [4] study reported the efficacy of docetaxel and prednisone regimen given as weekly (*n* = 334, 30 mg/m^2^) versus 3-weekly (*n* = 335, 75 mg/m^2^) cycles for the treatment of mCRPC. The PSA response (>50% decrease) in this study was 48% and 45% for weekly and 3-weekly docetaxel groups, respectively; the median duration of survival was 17.4 and 18.9 months, respectively. Malhotra and Poiezs retrospectively studied the weekly (30 mg/m^2^), 2-weekly (60 mg/m^2^), and 3-weekly (75 mg/m^2^) docetaxel regimens and reported PSA response rate in 58%, 71%, and 67% of the patients, with a median OS of 8.9, 23.3, and 16.3 months, respectively [25]. The PROSTY study group compared the 2-weekly (*n* = 170, 50 mg/m^2^) and 3-weekly (*n* = 176, 75 mg/m^2^) docetaxel regimens in a Phase III study for the treatment of mCRPC and demonstrated no significant difference in the PSA response rates between the regimens (2 weekly: 49% vs. 3 weekly: 42%; *P* = 0.486). Furthermore, for the 2-weekly and 3-weekly docetaxel regimens, the median TTF was 5.6 months vs. 4.9 months, with the median OS at 19.5 months vs. 17 months, respectively [26]. In our study, NDLS was administered as 2-weekly or 3-weekly regimens with PSA response observed in 66.7% (16/24) patients. The PSA response rate was 77.8% in the 2-weekly group vs. 60% in the 3-weekly group, which could be attributed to the higher actual dose intensity and higher cumulative median dose in the 2-weekly group.

The relative dose intensity for docetaxel in our study was 84% in the 2-weekly group and 75% in the 3-weekly group. The median OS was 14.6 months in the 2-weekly group whereas it was not reached in the 3-weekly group; the median TTF was 6.7 and 6.5 months, respectively, in this study. The effects of docetaxel dose-intensity on OS in patients with metastatic castration-sensitive prostate cancer demonstrated that reduced relative dose intensity was significantly associated with OS advantage (hazards ratio (HR): 1.18, 95% CI: 1.02–1.36, *P* = 0.026), and the risk of death increased by 23% (HR 1.23, 95% CI 1.09–1.4, *P* = 0.001) for every 10% decrease in relative dose intensity. [27].

Grade 3/4 AEs reported less frequently in the 2-weekly versus the 3-weekly group in the PROSTY study: neutropenia (36% vs. 53%), leucopenia (13% vs. 29%), and febrile neutropenia (4% vs. 14%). In the study by Melhotra and Poiezs, grade 3/4 AEs were reported in 8.3%, 28.5%, and 20% patients in the docetaxel weekly, 2-weekly, and 3-weekly groups, respectively [25]. Yoon et al. reported that 2-weekly docetaxel was generally well tolerated and alopecia (74%), nail changes (42%), and constipation (31%) were the most common AEs reported [7]. In our study, AEs such as peripheral neuropathy, fluid retention, and acute hypersensitivity reactions were not reported with NDLS chemotherapy, which are commonly reported with conventional docetaxel formulation [28]. Anemia, lymphopenia, thrombocytopenia, and neutropenia were the hematological AEs whereas nausea, weakness, constipation, vomiting, and diarrhea were the most common (≥10%) nonhematological AEs reported in our study. The hematological AEs were less in the 2-weekly group vs. the 3-weekly group despite higher median actual dose intensity in the 2-weekly group compared to the 3-weekly group (21.04 vs. 18.75 mg/m^2^/week). Grade 3 AEs were reported in the 2-weekly group (neutropenia and diarrhea in 2 patients each) but not reported in the 3-weekly group. None of the patients reported grade IV AEs.

Intas Pharmaceuticals Limited, India, has developed the NDLS formulation using lipids generally regarded as safe (GRAS) by the US Food and Drug Administration (USFDA) based on the patented ‘NanoAqualip' technology (patent numbers: worldwide (WO2008127358), Europe (2076244), Japan (5917789), and Canada (CA2666322)). NDLS formulation is devoid of polysorbate 80 and ethanol. For the development of NDLS, docetaxel is added to high-pressure homogenized soy phosphatidylcholine and sodium cholesteryl sulfate in sodium citrate buffer under continuous high-pressure homogenization [17]. The delivery of docetaxel may be increased to the tumor tissues with the resultant nanosomal (<100 nm) particles of NDLS and due to the damaged tumor vasculature, resulting in an enhanced permeability and retention effect. This can result in a greater systemic availability of docetaxel from NDLS formulation [17], and thus, improved therapeutic outcomes can be potentially expected [29]. In addition, polysorbate 80 and ethanol-related toxicity issues can be circumvented as well.

The study limitations included the retrospective nature of the study, a small sample size, and the lack of completeness of safety data. The progression-free survival (PFS) could not be captured in this study since, being a real-world study, the data on progression were not available for most of the patients at most of the follow-up time points.

## 5. Conclusions

Nanosomal docetaxel lipid suspension (NDLS) as 2-weekly and 3-weekly regimens was effective and well tolerated in managing patients with mCRPC. A prospective phase-4 clinical trial is underway (CTRI/2018/02/012212) to evaluate the safety and efficacy of NDLS in mCRPC.

## Figures and Tables

**Table 1:** Demographic and baseline characteristics.

Characteristics	2-weekly NDLS (*n* = 9)	3-weekly NDLS (*n* = 15)
Age (years), *n* (%)		
<65 years	2 (22.22)	7 (46.67)
65–74 years	3 (33.33)	4 (26.67)
≥75 years	4 (44.44)	4 (26.67)
Baseline BSA (median (range))^*∗*^	1.7 (1.5–1.9)	1.7 (1.2–1.9)
Median follow-up duration, months (range)	14.7 (5.5–25.7)	12.2 (7.9–15.6)
ECOG performance score, *n*		
0ss	5 (55.55)	3 (20)
1	3 (33.33)	8 (53.33)
2	1 (11.11)	2 (13.33)
3	0	2 (13.33)
Gleason score at initial diagnosis		
≤7	4 (44.44)	13 (86.67)
8	1 (11.11)	2 (13.33)
≥9	2 (22.22)	0
Unknown	2 (22.22)	0
Median PSA at baseline, (range), ng/mL	226 (18.17–510)	28 (1.6–2030)
Median baseline Hb (range)	10.9 (9.8–12.7)	10.8 (8.2–13.1)
Metastasis site		
Bone	7 (77.77)	15 (100)
Unknown	2 (22.22)	0
Previous therapy		
Radiotherapy	3 (33.33)	4 (26.67)
Prostatectomy	5 (55.55)	12 (80)
Orchiectomy	4 (44.4)	11 (73.3)
Previous systemic therapy		
Bicalutamide	0	4 (26.67)
Abiraterone	0	8 (53.33)
Comorbidities^*∗∗*^		
Diabetes	2 (22.22)	6 (40)
Hypertension	0	7 (46.67)

BSA = body surface area, ECOG = Eastern Cooperative Oncology Group, Hb = hemoglobin; NDLS = nanosomal docetaxel lipid suspension, PSA = prostate specific antigen. ^*∗*^Baseline BSA was not available for one patient who received 2-weekly NDLS. ^*∗∗*^Other comorbidities include tuberculosis, heart disease, asthma, and abdominal hernia.

**Table 2:** Treatment delivery.

Treatment	2-weekly NDLS (*N* = 9)	3-weekly NDLS (*N* = 15)
Cumulative dose (mg), median (range)	650 (240–1660)	500 (300–750)
No. of cycles, median (range)	14 (6–40)	10 (6–11)
Actual dose intensity (mg/m^2^/week), median (range)	21.04 (20–37.50)	18.75 (16.67–25)
Relative dose intensity^*∗*^ (%), median (range)	84 (80–150)	75 (67–100)

^*∗*^Calculated at a planned dose intensity of 25 mg/m^2^/week.

**Table 3:** Efficacy evaluation.

Parameter		2-weekly NDLS (*n* = 9) (%)	3-weekly NDLS (*n* = 15) (%)
PSA decline	PSA decline >50%	77.8%	60%
	PSA decline >90%	55.6%	40%
Median %PSA decline		96.31%	83.29%
Median TTF (days)		200	195
Therapy after NDLS treatment^∗^	Abiraterone (*n* = 4)	1	3
	Biculatamide^∗∗^ (*n* = 5)	0	5
	Cabazitaxel (*n* = 1)	1	0
	Cyclophosphamide (*n* = 1)	0	1
	Enzalutamide (*n* = 2)	1	1
	Mitoxantrone (*n* = 1)	0	1

NDLS = nanosomal docetaxel lipid suspension, PSA = prostate specific antigen; TTF, time-to-treatment failure. ^*∗*^Details for therapy after NDLS treatment are available for 14 patients only. ^*∗∗*^One patient received fosfesterol, and another patient received leuprolide along with bicalutamide who had received 3-weekly NDLS as second-line therapy.

**Table 4:** Safety profile.

AEs	2-weekly group (*N* = 9)		3-weekly group (*N* = 15)
	Grade I/II, *n* (%)	Grade III, *n* (%)	All grade I/II, *n* (%)
Hematological AEs			
Anemia	8 (88.89)	—	13 (86.67)
Lymphopenia	6 (66.67)	—	5 (33.33)
Thrombocytopenia	2 (22.22)	—	2 (13.33)
Neutropenia	3 (33.33)	2 (22.22)	—

Nonhematological AEs			
Nausea	1 (11.11)	—	4 (26.67)
Vomiting	1 (11.11)	—	6 (40)
Weakness	3 (33.33)	—	9 (60)
Hyperglycemia	1 (11.11)	—	—
Anorexia	—	—	1 (6.67)
Diarrhea	—	2 (22.22)	4 (26.67)
Alteration in LFT	—		1 (6.67)
Mouth ulcer	1 (11.11)	—	—
Constipation	2 (22.22)	—	6 (40)

AE = adverse event, LFT = liver function test, NDLS = nanosomal docetaxel lipid suspension.

## Data Availability

Datasets used in this analysis can be provided upon reasonable request from the authors.
